# Conservative management of spontaneous coronary artery dissection: a case report

**DOI:** 10.11604/pamj.2020.36.334.25546

**Published:** 2020-08-25

**Authors:** Hind Regragui, Badre El Boussaadani, Lamyae Sasbou, Hanae Bouhdadi, Hicham Wazaren, Mohammed Cherti

**Affiliations:** 1 Cardiology B Department of Ibn Sina University Hospital Center, Mohammed V University in Rabat, Rabat, Morocco; 2 Cardiovascular Surgery A Department of Ibn Sina University Hospital Center, Mohammed V University in Rabat, Rabat, Morocco

**Keywords:** Spontaneous coronary artery dissection, acute coronary syndromes, conservative management, case report

## Abstract

Spontaneous coronary artery dissection (SCAD) is a rare cause of acute coronary syndromes (ACS) that mainly occurs in young women with no risk factors and no coronary atherosclerosis. Diagnosis is made by invasive coronary angiography (CA), computed tomography coronary angiography (CTCA), intravascular ultrasound (IVUS) and optical coherence tomography (OCT). The rarity of this entity as well as the complications of invasive treatment make it difficult to choose therapy between conservative management, percutaneous coronary intervention (PCI) or coronary artery bypass grafting (CABG). We report a case of a 36-year-old woman presented with non ST elevation myocardial infarction (NSTEMI) related to spontaneous dissection of coronary arteries (left main trunk, left anterior descending artery and left circumflex artery) treated medically with spectacular results at 2 months, controlled by CTCA.

## Introduction

Spontaneous coronary artery dissection (SCAD) is considered as a cause of acute coronary syndromes (ACS) of uncertain origin that mainly occurs in young women [1]. The association with fibromuscular dysplasia or pregnancy is common [2]. Chest pain is the most common chief complaint of SCAD, and the diagnosis is made by invasive coronary angiography (CA), computed tomography coronary angiography (CTCA), intravascular ultrasound (IVUS) and optical coherence tomography (OCT). There are 3 therapeutic options including conservative medical treatment, stenting and surgical revascularisation by coronary artery bypass grafting (CABG), but there is no consensus on treatment of SCAD which makes it difficult to manage.

## Patient and observation

**Figure 1:**
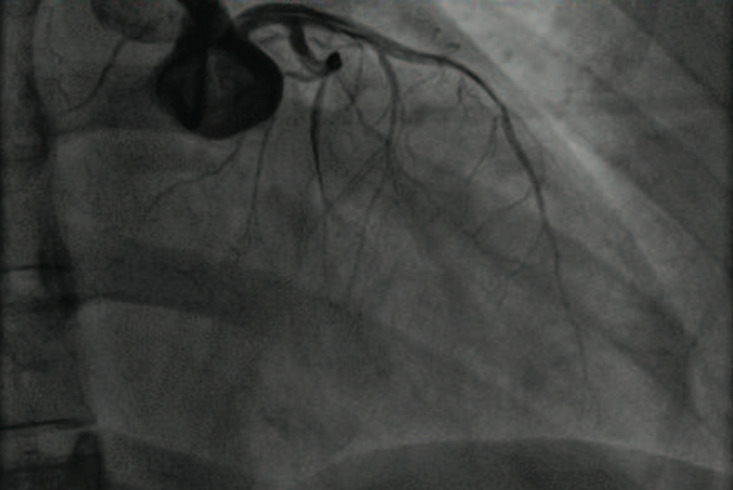
SCAD of LAD with extension to left main trunk and LCX at invasive coronary angiography

A 36-year-old woman, active smoker with history of an untreated dyslipidemia, was admitted to our department for a chest pain radiating in hemi-belt, in the two upper limbs and sometimes in the neck, occurring at rest, with an intermittent character and associated with nausea, palpitations, dyspnea and dry cough, without notion of fever or recent flu syndrome. The clinical examination being without abnormalities, an electrocardigram was performed and objectified diffuse microvoltage and fragmentation of the QRS complex in DIII and aVF leads with negative T wave in aVL lead. The troponin level was up to 8 times the normal value. At this step, the patient is diagnosed as a non ST elevation myocardial infarction (NSTEMI). At transthoracic echography (TTE), the left ventricle (LV) was undilated, non-hypertrophied with latero-apical dyskinesia, and LV ejection fraction (LVEF) at 50-55%, minimal central mitral regurgitation on normal mitral valves, normal right cavities, and there was no pericardial effusion. Coronary angiography (Figure 1) demonstrated an aspect evoking a spontaneous dissection of the distal left anterior descending artery (LAD) going back to the left main trunk as well as to the left circumflex artery (LCX). The flow on the LVA was thrombolysis in myocardial infarction (TIMI) grade 2-3 and slowed down at the level of the distal circumflex. The dissection was aggravated upon injection into the left main trunk with extensive dissection of the entire left coronary.

**Figure 2:**
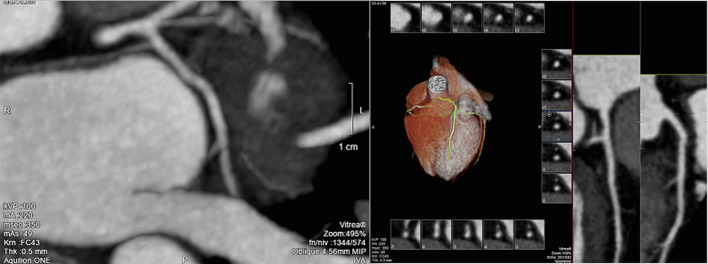
resolution of SCAD at CTCA: LAD view

**Figure 3:**
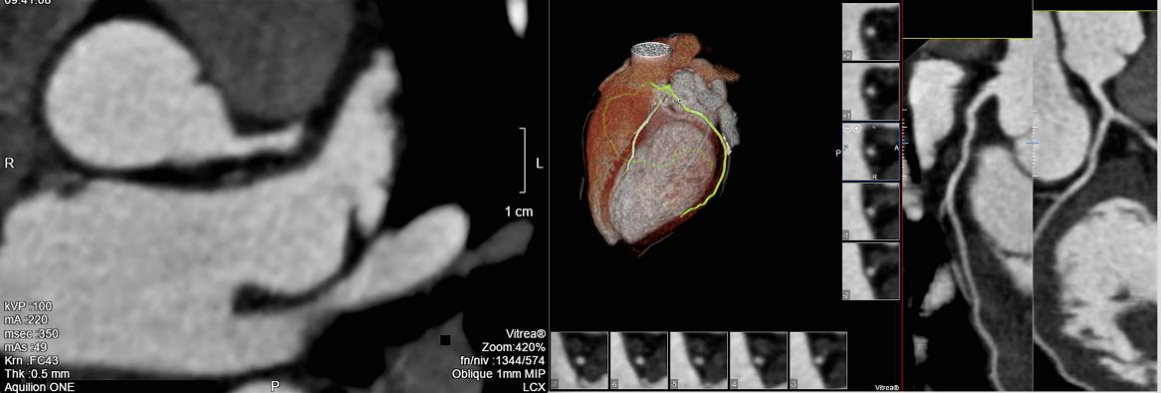
resolution of SCAD at CTCA: LCX view

The dissection was occlusive on the LCX and the flow was correct on the LAD. After a team consultation between intensive care cardiologists and cardiac surgeon, and considering clinical and hemodynamic stability, we decided to give medical treatment with a single antiplatelet agent without anticoagulant treatment because of the major risk of worsening the extension of the dissection by stenting. Urgent surgical revascularization does not appear to be desirable in this context of spontaneous coronary dissection either. We decided to respect the dissection, to treat with only aspirin and to keep the patient at the intensive care unit. The outcome was favorable, with progressive pain relief and a stable hemodynamic state. There was a clear elevation of troponin and creatine phosphokinase (CPK) levels, an inflammatory syndrome with considerable elevation of C-reactive protein and white blood cells, without evidence of bacterial infection, probably related to myocardial necrosis or viral disease, gradually regressive during hospitalization. On the rhythmic level, there was an initial ventricular hyperexcitability, leading to the introduction of beta-blockers with favorable course. The level of low-density lipoprotein (LDL) cholesterol in the blood was 1.80g/dl and atorvastatin was introduced. Discharge treatment included aspirin 75mg, atorvastatin 40mg and bisoprolol 5mg, and smoking was stopped. Computed tomography-coronary angiography (CTCA) realised after 2 months to assess healing showed a non-calcified coronary network and a complete resolution of the dissection (Figure 2, Figure 3).

## Discussion

SCAD remains a rare cause of acute myocardial ischemia. It occurs in middle aged women in 80% of cases and more than 25% of these are in the peri-partum period, in the absence of coronary atherosclerosis and risk factors [1]. Generally, patients who may develop a SCAD have predisposing factors such as fibromuscular dysplasia (FMD), female gender, pregnancy-related factors, possibly hormonal therapy, mixed connective tissue disorders and inflammatory disorders [2]. SCAD is the result of a separation within the coronary artery wall caused by intramural haemorrhage made up from the development of an endothelial and intimal discontinuity or tearing, or from the primary disruption of a vasa vasorum micro-vessel leading to haemorrhage directly into the media, creating a false lumen leading to compression of the true lumen [3]. The typical presentations are STEMI, NSTEMI or unstable angina, presenting with chest pain, ventricular arrhythmias, or sudden death [4]. Faced to the clinical symptomatology of chest pain with electrical changes, enzymatic movement of cardiac markers and echocardiography kinetic disturbances, the positive diagnosis of SCAD can be suspected especially in young women with no cardiovascular risk factors. At this stage, some differential diagnosis should be considered, especially in view of the rarity of this entity, namely Takotsubo cardiomyopathy, coronary artery spasm, coronary thromboembolism, atherosclerotic ACS [5].

Different methods can be used to make the diagnosis of SCAD. CTCA, frequently used to assess acute chest pain presentations, has the advantage of being non invasive. However, it is usually used after CA and in the follow-up due to the possibility of false negative findings [3]. Therefore, CTCA does not replace CA which remains the main test of choice for the diagnosis of SCAD. In addition, IVUS used to differentiate atherosclerotic plaques from SCAD, clearly shows the true and false coronary lumens and the extent of the false lumen thrombosis (3). Furthermore, OCT has the advantage of better spatial resolution and good characterization of images [3]. There is no consensus on the treatment of SCAD. Therapeutic possibilities are conservative medical treatment, PCI and CABG. Shamloo BK *et al*. [6], in their study of 440 cases, showed that patients with a single lesion in left or right coronary artery had a better outcome and less need for further intervention when treated with an early aggressive strategy, with either CABG or PCI, compared to a conservative strategy. On the other side,

Alfonso *et al*. [7], in their prospective study of 45 patients, suggested as first-choice a “watchful waiting” approach in stable patients, with a possible switch to revascularization in case of ongoing or recurrent ischemia.PCI is accompanied by a risk of adverse events including: extension of the dissection, guidewire passage into the false lumen and major side branch restriction or occlusion by propagation of haematoma [3]. A high rate of late bypass graft occlusion has been reported in patients who benefited from CABG, suggesting that CABG may not provide long-term protection against the effects of recurrent SCAD [4]. Therefore, CABG should be limited to patient with persistent or reccurent ischemic pain, after a PCI failure on main coronary arteries and for multivessel or left main SCAD [8]. Lettieri *et al*. [8] identified some clinical and angiographic features that might help in the decision-making process. For patients with NSTEMI or transient STEMI and basal TIMI grade 2 or 3 flow, especially in small and medium vessels, an initial conservative strategy apparently guarantees great immediate results. Otherwise, revascularization should be performed promptly for patients with large occluded vessels.

## Conclusion

In conclusion, conservative therapy seems to have excellent results and should be considered as first-choice therapy if the patient is clinically and hemodynamically stable even if major arteries are affected.
